# Traditional Individual and Environmental Determinants of Healthy Eating in Vihiga County, Western Kenya

**DOI:** 10.3390/nu14142791

**Published:** 2022-07-07

**Authors:** Daniela Penafiel, Celine Termote, Patrick Van Damme

**Affiliations:** 1 Research Center, Faculty of Medicine, Universidad de Especialidades Espíritu Santo, Samborondom 092301, Ecuador; 2 Rural Research Center, Faculty of Social Sciences, Escuela Superior Politècnica del Litoral, Guayaquil 090112, Ecuador; 3 Alliance of Bioversity International and CIAT, Via dei Tre Denari 472/a, 00054 Rome, Italy; c.termote@cgiar.org; 4 Faculty of Tropical AgriSciences, Czech University of Life Sciences, 165 00 Prague, Czech Republic; van_damme@frz.czu.cz

**Keywords:** diet, determinants of eating, environment, traditional foods, taboos

## Abstract

Traditional ethnic groups in Kenya are unlikely to eat a healthy and diversified diet due to many individual and environmental factors, which may result in poor health status. Therefore, the determinants of eating behavior need to be identified prior to any public health action. For this study, focus group discussions (15 in total) in a double-layer design were conducted, comprising adult men and women from 5 villages of Vihiga County. Questions explored knowledge; barriers and cues to action toward eating a healthy diet containing a variety of foods; including indigenous food species. We found that healthy eating concepts are known; however, several taboos that restrict food consumption reduce local diet quality in terms of diversity. Nutrition education is a cue to action. We identified several individual and environmental determinants of eating behavior in the studied communities. Public health action should focus on supporting healthy eating behaviors and refining some taboos’ beliefs.

## 1. Introduction

Worldwide, there is increasing interest in understanding human eating behavior, as it determines both healthy and unhealthy eating choices that ultimately affect people’s nutritional status [1]. Unhealthy eating can be considered as a risk factor for poor health which is often associated with the consumption of ultra-processed, salty and fatty foods. Western diets are defined (MeSH term) as “a pattern of food consumption adopted mainly by the people of North America and Western Europe which is mainly characterized by high intake of meat, processed grains, dietary sugars, dairy products and dietary fats”. Clearly, eating behavior differs between cultures, particularly between people living in western countries and traditional communities living in the developing world, due to their different environments (e.g., cultural backgrounds or food systems).

It is widely known that eating a diversity of foods promotes good health (ibid.). One might think that eating cultures developed in environments that have a high diversity of foods, may be influenced by the availability of it, but in reality diversified diets are determined by many other individual and environmental factors. 

Numerous developing countries aim to promote healthy eating behaviors that result in high Food Variety Scores (FVS) or Dietary Diversity Scores (DDS) because of the high association of these indicators with overall nutrient adequacy and nutritional status [2]. To increase dietary diversification, public health action needs to identify individual and environmental context-specific determinants that influence communities’ eating behaviors. Action research provides the latter evidence to the stakeholders involved in public health interventions. 

A wealth of evidence shows the association between several dietary diversity indicators and the nutrient adequacy ratio (NAR) in developing countries. In Mali, for example, children’s diets scoring 6 for DDS or 23 for FVS are nutrient-adequate, with DDS being a stronger predictor than FVS [3]. In South Africa, a DDS of 4 and FVS of 6 were found to be strongly associated with micronutrient adequacy and child growth [4]. A more recent study that analyzed data from 7 developing countries found an association between species richness (SR) and NAR and showed that eating a variety of food species is a good predictor of micronutrient adequacy for both children and women [5].

Likewise, high dietary diversity has been associated with reduced chances of being malnourished [6]. Unfortunately, the population’s poorest segments are the most affected by low dietary diversity (ibid). The double burden of malnutrition (both undernourished or overweight) is spread across generations from women of reproductive age who become mothers to their offspring [7]. Several studies have evidenced the importance of dietary diversification for children’s and women’s nutritional status [2,3,5,8,9].

In search of indicators to monitor dietary diversity in children and women, infant and young infant and child feeding minimum dietary diversity (YICDS) and minimum dietary diversity score for women of reproductive age (MDD-W) have been proposed as proxy indicators for monitoring micronutrient adequacy, using a cutoff of 4 good groups out of 7 for children and 5 food groups out of 10 for women [10]. These indicators have been used to identify populations that are highly likely to select at least one nutrient-rich food group, such as animal-flesh foods, eggs, vitamin A-rich fruits, and vegetables, that can supply the required micronutrients according to age group. Despite the growing body of evidence supporting dietary diversity as an indicator for nutrient adequacy, the behavioral factors of developing populations that influence eating a diet that contains a wide selection of food and food groups are still not fully understood.

The results from studies in developing countries evidencing low dietary diversity show that rural and urban African communities have monotonous diets that are traditionally based on starchy staples and limited protein-rich foods with limited servings of vitamin-rich fresh fruits and vegetables [2]. In this regard, a case study in the Democratic Republic of Congo showed that people living in biodiversity-rich environments, with ample traditional knowledge of species use, do not necessarily have a better diet [11]. More recently, a meta-analysis on dietary diversity in African farming systems showed that to increase the consumption of one food group, using the DD indicator, farmers would need to cultivate approximately 9 additional edible species on their farms [12]. More research is thus needed to determine the determinants of eating a diet that contains a wide variety of foods.

It is known that eating behaviors in African countries have been historically affected by many determinants, such as cultural preferences, poverty, income, socio-economic level, colonization, access to goods and services, taboos and others [13,14,15]. More specifically, Kenya is an example of an African country where low dietary diversity is affecting mainly children and women. A report on the health status of children and women of reproductive age in Vihiga County, located in Western Kenya, evidenced that they had DD scores lower than 4 [16].

Knowing that inducing behavior change requires a clear understanding of all the factors that influence a particular eating behavior, whether individual or environmental, we, therefore, analyzed the perceptions about having a healthy diet that is based on a variety of foods. In addition, we analyzed the perceived barriers to eating a variety of foods. Therefore, we conducted a qualitative investigation through focus group discussions (FGDs) to have participants talk about their perceptions in groups that are familiar to them. Our findings aim to inform policies that prevent unhealthy eating practices and promote healthy behaviors. 

Food Variety Score (FVS) is measured by counting the number of all foods consumed in a period of time (often 7 or 15 days). Dietary Diversity (DD) is measured by counting the number of food groups consumed in a day. Nutrient Adequacy Ratio (NAR) is calculated by dividing a person‘s nutrient intake by the Estimated Average Requirement (EAR) of the studied nutrient. Child Growth measured by Height by Age (H/A z-scores) and Weight by Age (W/A) z-scores. Species Richness (SR) is measured by counting the number of edible species consumed per day.

## 2. Materials and Methods

### 2.1. Study Area

This study was conducted in Vihiga County, and some tribes located in Western Kenya. According to the population and housing census held in 2009, it is known to have the highest population density in Kenya, apart from Nairobi, with a population density (1045 persons/km^2)^ and to be highly vulnerable to food insecurity. Additionally, according to the 2021 global nutrition report, the poor health status of the Kenyan population is reflected by the high prevalence of maternal anemia (28%) and child stunting (26%). Protein-energy malnutrition is the fourth main cause of death in Kenya, with more than 35,000 deaths in 2019. Additionally, 45% of children consume less than four food groups per day (mean DDS is 3.7 food groups per day out of 7 recommended food groups); likewise, women of reproductive age had an average DDSW of 4.2 (out of 9 food groups).

Over-nutrition is also present in Kenya, including the western part of the country, with a higher prevalence in girls (18%) than in boys (8%). This double malnutrition burden is increasingly manifesting itself in Kenya, similar to other developing countries, and causes high economic and health losses [7].

### 2.2. Action Research

The use of action research was necessary in this study to merge the most appropriate principles of behavioral research into findings that aim to improve the life quality of the studied group by involving them in the research process [17]. Using a collaborative approach resulted in a positive experience while achieving higher dietary diversification by our research group [18]. However, action can be motivated by traditions, emotions, values and beliefs which lingers strongly in the behavior of people and are context specific. Therefore, it is imperative that action research captures the perceptions of people who self-evaluate their cues to action aiming thereby collective benefits. By this practice, our research does not focus on the individual perception of a researcher or a participant but beyond a common idea is to document joint perceptions. 

### 2.3. Focus Groups Discussions

This study used focus groups to identify determinants of eating behavior in Vihiga County. FGDs are often used to interview people over a particular subject, generating responses and comments that come in the form of brainstorming and that are validated in the presence of the other participants, resembling a fluent conversation in familiar settings [19]. TFDs were selected over individual interviews because we wanted to analyze individual determinants that were validated by others and environmental determinants that are perceived as a group. In addition, in our field visits we identified that it is culturally appropriate to discuss topics about eating using group dynamics instead of individual visits which generate doubt about the study intentions. When participants were in a group they felt that all were treated equally. In addition, we have a group dynamic that was leaded by those eager to share and were followed by the shy ones. More importantly, the analysis of FGDs allows us to answer why and how to take action about the studied eating behavior, aiming to promote environmental support for action, such as nutrition education [20].

### 2.4. Study Design of FGDs

This study used a double-layer design (Table 1) to sample different villages of Vihiga County as the first layer and used demographic characteristics, i.e., gender and age range as the second layer, creating homogenous groups to study whether the area, gender or age can explain differences in the perceptions of eating a diversified diet. Respondents’ inclusion criteria consisted of (i) voluntary participation, (ii) being resident of Vihiga County, (iii) being adult (age > 18) and (iv) willingness to be interviewed in the absence of their spouse (assuming that women would respond differently in the presence of men).

Three group discussions were conducted in each of the 5 villages that were randomly selected out of the 10 villages that belong to Vihiga County (Ebunangwe, Emaloba, Emanda, Essunza, Itumbu, Mambai, Masana, Mwitubwi, Vigulu and Wanond). The list of villages used for randomization was provided by the “Humid Tropics CGIAR Research Project, crosscutting nutrition cluster—Western Kenya (2014–2016)” project. A total of 15 FGDs were needed to complete all the design layers (5 villages × 3 groups = 15 FGDs).

### 2.5. Theoretical Model to Study Eating Behavior

Throughout this study, eating behavior is defined as the “thoughts, actions and intentions that humans enact to ingest foods” [21]. In behavioral research, theoretical models of behavior are used to guide the research to explain and model a studied behavior. This study focused on three selected constructs (i.e., cognitive determinants of eating, perceived barriers, and cues to action) of the Health Belief Model (HBM), as it was shown to adequately capture determinants of eating a diverse diet in populations living in humid tropical environments [22].

### 2.6. Semi-Structured Questionnaire

To guide FGDs, a semi structured questionnaire (SSQ) was designed by all researchers, inspired by the 3 HBM constructs. Table 2 lists the questions used in the SSQ after revision, pretest and approval by the 3 authors. The pretest was piloted one week before FGDs were conducted under conditions similar to approved protocols. The moderator used the SSQ to guide the FGDs and when the main questions was not responded the list of alternative questions was used. The questions escalated from the perceptive level, starting with open questions that helped to frame ideas around the research topic. Then, the questions were elevated to cognitive questions asking concepts about healthy and diversified eating, followed by asking respondents about the barriers encountered to engage in eating healthy and diversified diets to further elevate the discussion up to proposing possible solutions to the listed barriers [23]. After asking open questions about ‘cues to action’, group interviews’ participants agreed to rank specific actions that they perceived to be the most important. The top 3 cues to action were identified by a ranking exercise that used an A3 paper listing all mentioned cues to action and beans that serve as beads to count the voting. 

### 2.7. Participation

Before conducting the FGDs, Bioversity International obtained ethical clearance for the whole project from the Egerton University ad hoc commission. Participants who met the inclusion criteria received a consent form explaining the purpose of the study and ensuring confidentiality. The consent form was in Swahili and read out loud to ensure that it was understood by everyone. Signed informed consent forms are stored at Bioversity International offices in Nairobi. Before using the SSQ, a sociodemographic questionnaire was provided to each participant to collect sociodemographic information.

On the day of the interviews, participants were gathered in a place that was familiar to them, where they were feeling confident (i.e., schools, health centers, communal places). All FGDs were held in Swahili and facilitated by a Kenyan moderator who is fluent in both English and Swahili and has a bachelor’s degree in nutrition science. Simultaneous translation from Swahili into English was provided by an experienced Kenyan translator with a bachelor’s degree in agriculture who was taking field notes in English during the group discussions, in this way the main ideas and the dynamics of each FGDs were ready to be analyzed by the main researcher right after each FGDs. Having a local translator from Vihiga County was an additional advantage to receive help on clarifying cultural practices that were related to diversification that are different in other continents. When new topics required clarification, these were introduced in the next focus group discussion using as a reference the name of the village where the topic was mentioned. The main researcher did not participate of the FGDs. To record all group discussions, we used a digital voice recorder (Philips, model LFH0635). After each group discussion, participants received a light lunch and transport allowance.

### 2.8. Content Analysis

Content analysis has been used as a qualitative interpretation technique since the beginning of the 20th century to answer research questions based on comments given by participants [24]. Qualitative content analysis of FGDs is recommended to explore eating behavior that is evident to the eye but has not been scientifically reported, which is the case for diversified eating behavior in Vihiga County. Interviews were transcribed into text (verbatim methods) from the original audios into English by a local translator using Word (Microsoft 10). Two transcripts were back-translated to ensure translation accuracy. The content of the transcripts was revised by the main researcher using the original FGD records and other sources that captured information such as field notes and photographs. Transcripts were labelled separately using the name of the village, age group and gender of the interviewed group participants and then imported into NVivo (QSR international—Melbourne, Australia—version 11.0).

The codes used to label the comments and for the analysis contained the 3 central constructs of the HBM (i.e., cognitive determinants, perceived barriers, cues to action), but we also created new free codes for additional findings [24]. All the created codes, also known as coding tree, were used as the heart of the qualitative content analysis.

Deductive analysis of the transcripts using the coding matrix was conducted based on representative inquiry, which allowed us to identify patterns of behavior reported by participants and evidenced by the analysis of the comments grounded on the selected theory (HMB) [25]. Using a theory is imperative for deductive analysis, particularly when aiming to identify determinants of behavioral change. Most of eating determinants reported in the literature have not used a theory or used a theory that does not explore behavioral change, therefore, and deductive approach has been chosen over an inductive one. Coding was facilitated by using the researchers’ comments, which are between parentheses throughout all verbatim transcriptions.

After the main researcher coded all transcripts, the other two field researchers confirmed the code given to each quotation to reduce bias, which in qualitative research is imperative to have internal validity and avoid that main researcher is not coding based on personal perceptions. Code confirmation was performed until an agreement was achieved, especially for quotes with double coding [26]. Since replication is not possible in social research, we used persistent observation to ensure that the researcher’s analysis truly reflects the participants’ behavior having thereby more credibility on our results [27]. The sociodemographic characteristics of the participants were used to summarize the sample. Descriptive statistics were calculated using RStudio to report means and standard deviations (SD) as well as percentages and frequencies for gender, marital status, education, occupation and religion.

## 3. Results

Table 3 gives an overview of the participants’ demographic characteristics. In total, 122 people participated in the FGDs, of whom approximately one-third were men and two-thirds were women. Participants’ ages ranged from 16 to 78, with a mean of 35.5 (12.8 SD). Studied participants reported having a mean number of children of 3.1 (2.3 SD). The majority (74.6%) of respondents were married, while almost everyone (99.2%) committed to the Christian faith. Approximately two-thirds had only attended primary school. Most of them did not have formal employment but rather considered themselves to be farmers (31.1%), housewives (27%), or either self- (11.5%) or unemployed (9.8%). 

There was no remarkable difference between villages in the determinants of eating a wide selection of foods, whereas taboos were mentioned to highly restrict the intake of animal-protein foods to children and women. Table 4 and Table 5, contain the most representative quotations for each determinant. 

### 3.1. Cognitive Determinants about Healthy Eating

#### 3.1.1. Concepts of Healthy Eating

Table 4 lists all cognitive determinants identified with some contestant’s quotes. Participants described their perception of healthy eating, mentioning that a good diet is composed of foods with all the nutrients needed by them. They said that eating different foods is a necessary condition to be healthy but that all nutrients are distributed in different meals over the day and over different foods. In general, healthy eating was linked to consuming a variety of foods, whereas unhealthy eating was associated with eating a monotonous diet. In all groups, eating fruits was mentioned as an essential determinant of healthy eating.

#### 3.1.2. Knowledge of Food Groups and Perceived Benefits

Participants mentioned that foods could be grouped according to their intrinsic composition, namely, foods rich in (i) carbohydrates, (ii) protein, (iii) vitamins, and also according to the origin: (iv) fruits and vegetables (plant origin).

Foods belonging to the carbohydrate group were mentioned to give energy to the body, for example, ugali, rice, cassava, and sweet and Irish potatoes. Foods supplying protein were mostly said to build body mass but also to provide energy, protect against diseases or increase blood quality; some examples include beans, fish, beef, milk, meat and chicken.

Foods rich in vitamins were said to prevent or protect against diseases, allowing local people to have a healthy life. Merging all quotations all 13 essential vitamins were mentioned by the participants (results not shown). Participants conceptualized food of plant origin merging fruits and vegetables together, but they classified traditional fruits and vegetables separately from conventional fruits and vegetables. The consumption of traditional fruits and vegetables was mentioned to have positive health benefits.

In general, healthy eating was also linked to other benefits, such as (i) helping to procreate, (ii) giving a feeling of happiness in their daily life, and (iii) organoleptic properties such as smell and flavor.

### 3.2. Environmental Determinants

#### Environmental Barriers to Eating a Diversified Diet

Environmental barriers include economic, physical barriers and political barriers because these involve external factors and not only the individual. 

The economic barrier toward the intake of a diverse diet referred to limitations to buying (i) a diverse food basket, (ii) food items that have a high cost, or (iii) inputs used to cultivate a variety of foods. Most participants mentioned that people with adequate financial means have a higher chance of consuming a variety of healthy foods because they can either buy them or cultivate a variety of crops on their land. However, a few respondents commented that money alone is not an essential element to have access to healthy foods, as they perceive that being wealthy is a necessary but not sufficient condition to have a balanced diet. 

In this line, many respondents agreed that having financial access goes in hand with having adequate knowledge about healthy eating that guides healthy eating choices and incorporates a variety of healthy foods into the diet. 

Physical barriers include the (i) limited area of land that is adequate to cultivate different crops and fruits and vegetables, (ii) lack of availability of different foods due to seasonality or regionality, (iii) climate change affecting the production and harvest of some cultivars, and (iv) fresh food markets located far from home. Table 5 lists all environmental determinants and quotes that are associated with the factors in the environment that affect the dietary diversity of the studied population. Respondents had very long conversations about this level of influence compared to the individual factors.

**Table 5:** Environmental determinants of healthy eating and quotations from participants of Focus Group Discussions in Vihiga County, Kenya.

Determinant	Quotations
Economic barrier	*“Money is the major problem. You can only buy the food if you have money.”* [W, 26] *“Lack of money will make one not to get the foods.”* [M, 70] *“You like to eat rice but there is no money. Money is the major problem.”* [W, 28] *“The foods are very expensive. We cannot afford it with the money we have.”* [M, 28] *“Prices are high … fruits you cannot afford when you don’t have enough money*.” [W, 23]
Physical barrier	*“Our farms are small. Cannot have all the mix of all foods in one farm.”* [W, 45] *“The farms are too small to plant varieties. There is no space for keeping domestic animals.”* [M, 44] *“Money can be available, but the food is not available on the market.”* [M, 22] *“There are seasons where we have food and other seasons where we don’t have because of the climate change.”* [M, 28] *“During the dry season, you may plant different foods, but they will not grow well to give you enough. And, also during the rainy seasons, you will not get enough because some foods don’t like too much rain.”* [W, 52] *“The markets are too far. … you have to go to Kisumu to purchase something”* [W, 26]

Coding between square brackets refers to the participant [ ], **M** for man, **W** for woman and the **number** for the age.

### 3.3. Individual and Socio-Cultural Barriers of a Diversified Diet 

Individual barriers include the (i) physical and mental health status of the person, (ii) lack of knowledge about healthy eating, and (iii) negative attitudes toward eating a diverse meal. Sociocultural barriers to eating a diverse diet included (i) families with numerous members who compete for foods, (ii) internal family conflicts such as domestic stress, (iii) limited nutrition education available, and (iv) land at risk for stealing crops. Table 6 contains the quotes for these determinants. 

Participants mentioned that a diverse diet could not be eaten by people who have a deficient digestive system, such as having allergies or intolerances associated with the consumption of a certain food, or by people with physical limitations who are not able to prepare their meals. Many participants mentioned that they felt they had limited knowledge about healthy food selection, cooking and consumption. Negative attitudes about healthy eating were more frequently observed in others and not in themselves; for example, neighbors who ignore using traditional foods such as vegetables and who are too lazy to cultivate foods even when they have land.

More often, barriers to eating at the individual level were associated with food taboos (listed in Table 7), which mainly restrict the consumption of animal protein by vulnerable people (i.e., children and women). Taboos were associated with a specific food and age group.

### 3.4. Cues to Action

The top 3 factors that would encourage respondents to step up toward the intake of diverse foods in meals include interventions to increase (i) employment, (ii) inputs for agriculture, and (iii) nutrition education and training for cultivation.

Employment was ranked as the top 1 cue for action by 14 out of the 15 FGDs. Having a job was perceived as helpful in eating different foods when considering that stable high-income jobs (formal and permanent contracts) are linked to earning more money that allows buying a variety of foods all year long, as opposed to low-income jobs, often casual jobs.

Working in agriculture was perceived as a permanent job because they were hired by farm owners to run food production. The second factor, ranked by 10 out of the 15 FDGs, was having higher access to agricultural inputs such as fertilizer, seedlings, tools, equipment and even land. The third cue to action, also ranked by 10 out of 15 FGDs, was in relation to increased educational interventions on how to practice self-sufficient agriculture and healthy eating to adults and children, respectively. Training to increase agricultural production was the principal factor necessary to eat a variety of foods. This latter was associated with the need for extension agents who are experts in nutrition-sensitive agriculture, meaning that they will recommend cultivating foods that are healthy and rich in nutrients. Transportation to reach training sites or for extension agents to reach farmers was specifically mentioned. Participants also highlighted that nutrition education should be organized by the government as part of the school curriculum, particularly in elementary and secondary schools. Additionally, they mentioned that the social influence of healthy peers who cultivate and eat healthy foods would be useful to involve young generations in healthy eating practices.

Cues to action with a lower ranking include solutions to local issues such as (i) having adequate infrastructure, i.e., shops, markets or health centers, (ii) having a better water supply, such as irrigation systems, (iii) receiving family planning to have fewer children who might compete for foods, (iv) receiving food aid for difficult situations and free school meals, (v) having access to loans to reactivate agricultural activities and other enterpernourishment, (vi) having support groups according to gender and agricultural activity, (vii) receiving permanent follow-up to agricultural production or nutritional interventions, (viii) improving local safety to avoid that final harvest becoming stolen, and (ix) empowering communities to work together and to engage in agriculture. Some quotes about all cues to action are:

“*money we get from the casual labor … not be enough to … save, because the work is not there all the time*.”

[W, 36]

“*If we can get somebody to help us to get fertilizers to use for planting, we will harvest enough*.”

[W, 49]

“*Seedlings should be provided to put in practice what we have learned*.”

[W, 32]

“*If we can get farms outside our area and we will plant different foods*.”

[M, 40]

“*… educated on new methods of farming*.”

[W, 40]

“*… on how to use the local manure other than the fertilizers*.”

[W, 42]

“*when keeping chicken, we should be educated on which foods to give to them*.”

[M, 19]

“*how to create our own manure from the resources we have, for example maize stalks*.”

[W,49]

“*… we have the food but we need to know what to do with it. It should be given nutrition education*.”

[W, 29]

“*Polytechnics to train the youth in the nearby small towns*.”

[M, 44]

“*Education should be provided by the government to children who want to learn*.”

[W, 37]

“*One can educate one another on diet education. One who is aware should educate the one who doesn’t know*.”

[W, 22]

## 4. Discussion

Our results show that several factors determining the eating behavior of people in Vihiga County can be improved either at the individual level (i.e., increasing knowledge and addressing taboos) or the sociocultural environment (i.e., through the provision of inputs for agriculture).

Since this study provides with evidence for action research, the scenario for healthy eating interventions in the study area seems optimistic since respondents have shown to have positive perceptions about eating a balanced diet, often linked to nutrient intake and eating frequency, which are important diet quality indicators in developing countries [11,22]. Likewise, healthy eating is associated with consuming fruits and vegetables, which are universal elements of a healthy diet and are used in Kenya as an indicator for optimal dietary composition [28]. More importantly, interventions should focus on promoting the consumption of traditional foods, as they are imperative for Kenya‘s dietary diversity [29]. In this line, our study is novel in that we have documented the perception of adult men in relation to dietary diversification which was not carried out by a previous study in Kenya that only targeted women and their children [18].

However, local stakeholders must be cautious when designing interventions. Our findings indicate that there are several barriers, mainly environmental barriers, that restrict the free selection of foods that hamper healthy and diversified eating. During our analysis, we observed that participants blamed the environment more than their behavior. Not surprisingly, employment is perceived as the solution to reach ideal dietary diversification, particularly because it is associated with having income and therefore financial access to buy different foods. The need for employment and income has been repeatedly reported in developing countries as a key element for improving diet quality [22,29,30,31,32]. A deeper analysis, however, recognizes that there are remarkable differences between having steady employment that generates revenues from agricultural trade and the income derived from a salary received from doing precarious farming activities, with the former being the desired income-generating activity. Despite some of our participants mentioning that eating healthy is predominant in high socioeconomic groups, the association of higher income with eating a higher number and amount of fruits and vegetables is only theoretical with limited causal evidence [33].

Receiving donations of agricultural inputs (i.e., fertilizers, seedlings, equipment) or loans to purchase these is a potential intervention that is supported by evidence that shows the positive relationship between land size and crop diversification [34,35]; however, diversity per hectare must be considered [36]. In addition to agricultural inputs, it is recommended to educate on how to cultivate different species but also how to cook or prepare these.

When focusing on cognitive barriers, we found that having limited knowledge about healthy food selection was perceived as a barrier when choosing among food groups, which is consistent with the overall, however weak, positive association between fruits and vegetable intake and knowledge [37,38].

The striking finding of our research is that current food taboos are still present and restrict the consumption of protein-rich foods, as was found 20 years ago [39], harming children aged below 2 and women of reproductive age who are paradoxically the most vulnerable to protein-energy malnutrition [40]. The interviews with women, in the absence of men, allowed us to identify that food taboos in Vihiga County are privileging adult men to eat animal protein, thereby neglecting the needs of vulnerable members of the household who have been culturally silenced. A previous study in India reported that ignorance and poverty and illiteracy are the leading determinants explaining the existence of wrong beliefs or misconceptions regarding foods directly associated with protein-energy malnutrition [41].

The existence of food taboos that restrict the intake of protein-rich foods is frightening the dietary diversity in Vihiga County, particularly because eggs and flesh foods count as one point (each) to the dichotomous indicators for young children and women of reproductive age [10].

Based on our findings, we recommend that a nutrition-sensitive agricultural intervention that combines agricultural and nutrition training toward dietary diversification is needed in the study area. The establishment of such intervention is supported by the Kenyan Nutrition Action Plant (2012–2017) and its nutrition-related objectives. These latter include supporting the education of topics such as complementary feeding, use of locally available energy- and protein-rich foods, cooking workshops, and kitchen hygiene (among others) toward improving the nutritional status of under-five children. However, it does not specifically address the education needed to reduce food taboos that restrict children from eating protein-rich foods. In this line, the findings of this action research are imperative to design future interventions. In general, some studies have reported that from 1974 until 2016, food taboos restricted the consumption of protein-rich foods supporting our findings [42,43]. In addition, addressing food taboos has been identified as a strategy to reduce maternal and child mortality in Indonesia as well [44].

Beyond the need for employment, agricultural inputs, and education, marketplace infrastructure is also required to increase the availability of a diversity of foods. The link between limited market access and household food insecurity is widely recognized. Sibhatu and colleagues (2015) found that distance to the market was negatively associated with dietary diversity in Ethiopia and Malawi [12].

The strength of our study is that we have some of the necessary information to start with the design of nutrition education educational messages. This is potentially possible because we (i) integrated three constructs of the HBM under a behavioral theory as framework, (ii) combined FGDs with participatory ranking to know the top cues to action, and (iii) identified specific food taboos to be addressed by educational messages. However, we still lack information about the causality of taboos and about effective interventions that have targeted similar eating patterns limiting protein intake. A limitation of our study is that our results are not empirically generalizable and do not apply to a population different from the studied one.

Future research should explore the origin of the taboos and reason these continue to be practiced. Additionally, the remaining constructs of HBM on food taboos, namely, the severity and susceptibility of suffering protein-energy malnutrition, the threat to health by not eating eggs and flesh foods by vulnerable groups (age, gender, ethnicity), and the perceived benefits of consuming protein-rich foods should be studied.

## 5. Conclusions

This study reports the determinants, both from people or the environment, of eating a diversified diet in Vihiga County (Western Kenya), which resulted in being cognitive, taboos restricting foods, and environmental barriers. Cues to action include support for nutrient-sensitive agriculture and education that guide the cultivation of a wide variety of foods. Remarkably, food taboos hamper the intake of protein-rich foods by restricting the count of one or two groups when using YICDS and MDDW indicators in children and women, respectively. Our results should be observed with caution as they do not represent the eating behavior of other Kenyan communities apart from the sampled ones. This study, however, serves as reference for future research that aims exploring behavior change using the HBM.

## Figures and Tables

**Table 1:** Double-layer design for Focus Group Discussions (FGD) in Vihiga County, Kenya.

Layer 1 (Villages)	Layer 2 (Gender-Age Range)
Essunza	Men > 18 Women ≤ 30 Women > 30
Itumbu	Men > 18 Women ≤ 30 Women > 30
Mambai	Men > 18 Women ≤ 30 Women > 30
Masana	Men > 18 Women ≤ 30 Women > 30
Wanondi	Men > 18 Women ≤ 30 Women > 30

**Table 2:** List of questions included in the Semi Structured Questionnaire for Focus Group Discussion in Vihiga County.

Question’s Goal	Determinant	Main Question	Alternative Question *
Introduction of participants	Food preferences	What is your name and what is your favorite food?	What is your name and where do you come from?
Exploration of cognitive determinants	Knowledge about healthy eating	How would you describe a good diet?	How would you describe a bad diet? Which foods would you consider to be healthy?
	Knowledge about food groups	How would you group the foods you know as healthy?	Which food group(s) do you consume more often? Which food groups do you consume in more amounts?
Exploration of individual barriers	Individual barriers	Why people do not eat a variety of foods?	Why cannot freely select a variety of foods for your meals?
		What stops you from freely eating a certain food or food groups?	Which foods cannot be freely eaten by you or other members of the family? What would happen if that person would eat that specific food?
Exploration of environmental barriers	Environmental barriers	Which people, apart from you, stops you from freely selecting a variety of foods to eat in a meal?	Why cannot you freely select from a wide variety of foods to eat in each meal?
Brainstorm on cues to action	Facilitators of change	What would help you to eat more diverse?	How can others help you to achieve dietary diversity?
Raking of cues to action	Top facilitator	Which are the 3 most important facilitators	

* alternative questions resulted from the pre-test of the SSQ and were used when participants requested to clarify the question.

**Table 3:** Demographic characteristics of the focus group discussion participants.

Characteristics	Total *n* (%)
**Gender** Male Female	39 (32) 83 (68)
**Marital Status** Married Single Widowed	91 (74.6) 24 (19.7) 7 (5.7)
**Education** Primary (incomplete) Primary Secondary University	28 (23) 48 (39.3) 36 (29.5) 10 (8.2)
**Occupation** Farmer Housewife Formal Employment Self-employed Casual worker Unemployed Other	38 (31.1) 33 (27) 7 (5.7) 4 (11.5) 4 (3.3) 12 (9.8) 14 (11.5)
**Religion** Christian Muslim	121 (99.2) 1 (0.8)

**Table 4:** Cognitive determinants of healthy eating and quotations from participants of Focus Group Discussions in Vihiga County, Kenya.

Determinant	Quotations
Concepts of healthy eating	*“Food that has the complete nutrients that make my day complete.”* [M, 56] *“For example, breakfast you can take tea with milk with sweet potatoes and finish with a fruit. Lunchtime, I mostly use porridge either maize meal or wimbi mixed with cassava. Then, at night you can eat ugali and chicken if it is available.”* [M, 19] *“Food should not be of only one type, but of different types.”* [M, 56] *“You have to change foods … when you make porridge and ugali it is the same food. You can change to tea with milk, at lunch you eat beans and in the evening you eat ugali. And you add a fruit.”* [W, 45]
Knowledge of food groups	*“It [Carbohydrate] adds energy to the body.”* [M, 40] *“Foods that build the body, the food that has proteins.”* [W, 40] *“Vitamins give good health. Fruits like avocado, oranges they have good health”* [W, 28] *“Good food is the one that protects the body from diseases.”* [M, 40] *“Ripe bananas help in digestion.”* [W, 24] *“… spinach helps in clearing the stomach for digestion.”* [W, 30] *“Managu helps the stomach for digestion.”* [W, 25] *“Green vegetables are good because they add blood and it is good.”* [W, 30] *“When I eat ugali and traditional vegetable they add energy. It helps me to do digging because it is stronger compared when I have eaten rice. This is good food.”* [M, 45] *“Ugali stays long in the stomach and I can do a lot of work because of the energy. And I can stay long without feeling hungry.”* [W, 29]
Benefits of healthy eating	*“Food that makes us happy … one that helps you carry family chores especially the married ones, you perform well in bed.”* [M, 38] *“Food with a good taste is a good food.”* [W, 29] *“Food that has a good appearance, that attracts to eat.”* [W, 32] *“Also the smell of the food, the aroma.”* [M, 28] *“Food should not be of only one type, but of different.”* [M, 56] *“You change foods … when you make porridge and ugali it is the same food. You can change to tea with milk, at lunch you eat beans and in the evening, you eat ugali. And you add a fruit.”* [W, 45]

Coding between square brackets refers to the participant [ ], **M** for man, **W** for woman and the **number** for the age.

**Table 6:** Individual determinants of healthy eating and quotations from participants of Focus Group Discussions in Vihiga County, Kenya.

Determinant	Quotations
Intrinsic barriers	*“Diseases, for example, ulcers. You are told not to eat for example sukuma wiki, githeri.”* [W, 26] *“Many disabled people cannot get food as they could wish.”* [W, 21] *“Most parents have no knowledge of a balanced diet; they should be educated.”* [M, 25] *“Lack of knowledge. Someone has enough but doesn’t know how to mix.”* [W, 31] *“For example, for the traditional vegetables some people tend to ignore eating them.”* [W, 40] *“It’s laziness. Women should work hard on the farm. They should plant some foods other than buying everything from the market.”* [W, 53]
Socio-cultural barriers	*“One has no job but has ten children. … Children will compete for food because there is not enough.”* [M, 44] *“Lack of peace in the house. I cannot cook when I am annoyed.”* [W, 25] *“It is also lack of education. … make one know how to eat different foods.”* [M, 45] *“Theft cases. Thieves steal the foods from the farms when it’s ready.”* [W, 32]
Taboos	*“The gizzard is supposed to be eaten by the men of the house, because the man is the household head. It is a sign of respect to the man.”* [M, 54] *“The gizzard should not be eaten by women, it’s for men.”* [W, 45] *“Gizzard is the sweetest part of the chicken. That is why they were denied.”* [M, 19] *“Children are not supposed to eat the gizzard because they will be competing with their fathers.”* [M, 24] *“When you take it, you become like the owner of the home and we are not supposed to be like the head of the home.”* [W, 37] *“Even up to date the children cannot eat the part of the gizzard. That one is serious.”* [M, 38] *“Backbone of chicken. It’s a respect to the men.”* [W, 27] *“The backbone has a lot of fats and it’s very sweet. They just don’t want women to be stronger than the men.”* [W, 50] *“Small kids, uncircumcised kids, should not eat chicken legs … when they go for circumcision, blood will come out too much.”* [W, 37] *“Women who have given birth cannot eat any type of fish until the baby is six months … body will get cracks that remove water.”* [W, 50] *“Pregnant women should not eat meat from a rabbit. Otherwise, they will give birth to a child who sleeps a lot.”* [W, 37] *“Lactating mothers should not eat meat from the rabbit. It will dry the breast milk. You will not have milk to breastfeed.”* [W, 29] *“… small children under the age of 2 years, they should not eat eggs, otherwise it will affect their speeches. Their tongue will become heavy and they delay to talk.”* [M, 38]

Coding between square brackets refers to the participant [ ], **M** for man, **W** for woman and the **number** for the age.

**Table 7:** Food taboos restricting the consumption of animal-protein foods reported in Vihiga County, Kenya.

Restricted Food	Affected Group	FGDs	Negative Beliefs
Chicken Gizzard Backbone Legs Eggs	Children < 2 years Women Women Boys through circumcision Children < 2 years	7 8 5 5 10	Children disrespecting fathers Women get empowered at household Women get stronger than men Excessive bleeding Delayed child’s talking skills
Fish	Lactating women	4	Reduced milk production; Skin irritation; Dehydration
Rabbit	Pregnant and lactating women	4	Offspring having bad manners; Child with excessive sleep; reduced milk production
Omena	Lactating women	3	Reduced milk production; Skin irritation

## Data Availability

The data that support the findings of this study are openly available in figshare at https://doi.org/10.6084/m9.figshare.13554452. Data was posted on 11 January 2021.

## References

[1] Thomson C.A., Foster G., Riekert K.A., Ockene J.K., Pbert L. (2014). Dietary behaviors: Promoting healthy eating. The Handbook of Health Behavior Change.

[2] Ruel M.T. (2003). Operationalizing dietary diversity: A review of measurement issues and research priorities. J. Nutr..

[3] Hatloy A., Torheim L.E., Oshaug A. (1998). Food variety a good indicator of nutritional adequacy of the diet? A case study from an urban area in Mali, West Africa. Eur. J. Clin. Nutr..

[4] Steyn N.P., Nel J.H., Nantel G., Kennedy G., Labadarios D. (2006). Food variety and dietary diversity scores in children: Are they good indicators of dietary adequacy?. Public Health Nutr..

[5] Lachat C., Raneri J.E., Walker K., Kolsteren P., Van Damme P., Verzelen K., Penafiel D., Vanhove W., Kennedy G., Hunter D. (2018). Dietary species richness as a measure of food biodiversity and nutritional quality of diets. Proc. Natl. Acad. Sci. USA.

[6] Arimond M., Ruel M.T. (2004). Dietary diversity is associated with child nutritional status: Evidence from 11 demographic and health surveys. J. Nutr..

[7] Delisle H. (2008). Poverty: The double burden of malnutrition in mothers and the intergenerational impact. Ann. N. Y. Acad. Sci..

[8] Moursi M.M., Arimond M., Dewey K.G., Tre S., Ruel M.T., Delpeuch F. (2008). Dietary diversity is a good predictor of the micronutrient density of the diet of 6- to 23-month-old children in Madagascar. J. Nutr..

[9] Onyango A., Koski K.G., Tucker K.L. (1998). Food diversity versus breastfeeding choice in determining anthropometric status in rural Kenyan toddlers. Int. J. Epidemiol..

[10] Food and Agriculture Organization of the United Nations (FAO), Family Health International (FHI 360) (2016). Minimum Dietary Diversity for Women: A Guide for Measurement. http://www.fao.org/3/a-i5486e.pdf.

[11] Termote C., Bwama Meyi M., Dhed’a Djailo B., Huybregts L., Lachat C., Kolsteren P., Van Damme P. (2012). A biodiverse rich environment does not contribute to a better diet: A case study from DR Congo. PLoS ONE.

[12] Sibhatu K.T., Krishna V.V., Qaim M. (2015). Production diversity and dietary diversity in smallholder farm households. Proc. Natl. Acad. Sci. USA.

[13] Meyer-Rochow V.B. (2009). Food taboos: Their origins and purposes. J. Ethnobiol. Ethnomed..

[14] Wambiya E.O.A., Kisia L., Osindo J., Kisiangani I., Kipruto S., Ilboudo P.G., Donfouet H.P.P., Mohamed S.F. (2021). Patterns and determinants of eating healthy in Kenya. Res. Sq..

[15] Steyn N.P., Nel J.H., Parker W.-A., Ayah R., Mbithe D. (2011). Dietary, social, and environmental determinants of obesity in Kenyan women. Scand. J. Public Health.

[16] Biodiversity International (2012). Improving Nutrition through Increased Utilisation of Local Agricultural Biodiversity in Kenya—The INULA Initiative. https://www.bioversityinternational.org/fileadmin/user_upload/online_library/publications/pdfs/Improving_nutrition_through_increased_utilisation_of_local_agricultural_biodiversity_in_Kenya_the_INULA_initiative_1815.pdf.

[17] McTaggart R. (1994). Participatory action research: Issues in theory and practice. Educ. Action Res..

[18] Boedecker J., Odhiambo Odour F., Lachat C., Van Damme P., Kennedy G., Termote C. (2019). Participatory farm diversification and nutrition education increase dietary diversity in Western Kenya. Matern. Child Nutr..

[19] Swift J.A., Tischler V. (2010). Qualitative research in nutrition and dietetics: Getting started. J. Hum. Nutr. Diet..

[20] Contento I.R. (2015). Nutrition Education: Linking Research, Theory, and Practice.

[21] Achterberg C., Clark K.L. (1992). A retrospective examination of theory use in nutrition education. J. Nutr. Educ..

[22] Penafiel D., Termote C., Lachat C., Espinel R., Kolsteren P., Van Damme P. (2016). Barriers to eating traditional foods vary by age group in Ecuador with biodiversity loss as a key issue. J. Nutr. Educ. Behav..

[23] Krau S.D. (2011). Creating educational objectives for patient education using the new Bloom’s Taxonomy. Nurs. Clin..

[24] Hsieh H.-F., Shannon S.E. (2005). Three approaches to qualitative content analysis. Qual. Health Res..

[25] Cho J.Y., Lee E.H. (2014). Reducing confusion about grounded theory and qualitative content analysis: Similarities and differences. Qual. Rep..

[26] Korstjens I., Moser A. (2018). Series: Practical guidance to qualitative research. Part 4: Trustworthiness and publishing. Eur. J. Gen. Pract..

[27] Pilnick A., Swift J. (2011). Qualitative research in nutrition and dietetics: Assessing quality. J. Hum. Nutr. Diet..

[28] Global Nutrition Report (GNR) (2021). Global Nutrition Report Country Profiles: Kenya. https://globalnutritionreport.org/resources/nutrition-profiles/africa/eastern-africa/kenya/.

[29] Ekesa B., Walingo M., Abukutsa-Onyango M. (2009). Influence of agricultural biodiversity on dietary diversity of preschool children in Matungu division, western Kenya. Afr. J. Food Agric. Nutr. Dev..

[30] Verstraeten R., Van Royen K., Ochoa-Avilés A., Penafiel D., Holdsworth M., Donoso S., Maes L., Kolsteren P. (2014). A conceptual framework for healthy eating behavior in Ecuadorian adolescents: A qualitative study. PLoS ONE.

[31] Verstraeten R., Leroy J.L., Pieniak Z., Ochoa-Avilès A., Holdsworth M., Verbeke W., Maes L., Kolsteren P. (2016). Individual and environmental factors influencing adolescents’ dietary behavior in low- and middle-income settings. PLoS ONE.

[32] Smart J.C., Tschirley D., Smart F. (2020). Diet quality and urbanization in Mozambique. Food Nutr. Bull..

[33] Kamphuis C.B.M., Giskes K., De Bruijn G., Wendel-Vos W., Brug J., Van Lenthe F.J. (2006). Environmental determinants of fruit and vegetable consumption among adults: A systematic review. Br. J. Nutr..

[34] Rehima M., Belay K., Dawit A., Rashid S. (2013). Factors affecting farmer’ crops diversification: Evidence from SNNPR, Ethiopia. Int. J. Agric. Sci..

[35] Sichoongwe K., Mapemba L., Tembo G., Ng’ong’ola D. (2014). The Determinants and extent of crop diversification among smallholder farmers: A case study of southern province Zambia. J. Agric. Sci..

[36] Pretty J., Toulmin C., Williams S. (2011). Sustainable intensification in African agriculture. Int. J. Agric. Sustain..

[37] Spronk I., Kullen C., Burdon C., O’Connor H. (2014). Relationship between nutrition knowledge and dietary intake. Br. J. Nutr..

[38] Guillaumie L., Godin G. (2010). Psychosocial determinants of fruit and vegetable intake in adult population: A systematic review. Int. J. Behav. Nutr. Phys. Act..

[39] Latham M.C. (1997). Human Nutrition in the Developing World.

[40] World Health Organization (WHO), Food and Agriculture Organization of the United Nations (FAO) (2002). Diet, Nutrition and the Prevention of Chronic Diseases.

[41] Das S. (2016). Textbook of Community Nutrition.

[42] Oniang’o R.K., Komokoti A. (1999). Food Habits in Kenya: The Effects of Change and Attendant Methodological Problems. Appetite.

[43] Ogbeide O. (1947). Nutritional hazards of food taboos and preferences in Mid-West Nigeria. Am. J. Clin. Nutr..

[44] Triratnawati A., Ditya Kristianti R., Putra A.P., Bagas Setyaji P. (2016). The effort to decrease maternal and child mortality rates through cultural transformation. Int. J. Public Health Sci..

